# Medical Opinion and Movement

**Published:** 1908-11-28

**Authors:** 


### Medical Opinion and Movement.

MR. G. M. BERINGER has written several papers in the American *Journal of Pharmacy* and elsewhere drawing attention to practical points that are of interest to medical men as well as to pharmacists. Amongst such points is the value of acetone as a solvent for cantharides. The active principle in the latter is present in part free, in part as a combined cantharidate. The latter is insoluble in chloroform and ether and alcohol. Cantharidin itself is more soluble in acetone than it is in other ordinary solvents, 1 part being dissolved by 38 of acetone, 66 of chloroform, 150 of acetic ether, or 700 of ordinary ether; it is still less soluble in alcohol. The officinal process for making collodion of cantharides requires that the powdered drug shall be percolated with chloroform, which means that only the free cantharidin is extracted. Mr. Beringer recommends the substitution of acetone for chloroform in the extraction of cantharidin, and of acetone for ether in the preparation of collodions; his formula for the latter is: cantharides, in fine powder, 60 grams; glacial acetic acid, 5 c.s.; pyroxylin, 4 grams; camphor, 1 gram; acetone, a sufficient quantity to make 100 grams. The point of chief practical interest is that the glacial acetic acid liberates the combined cantharidin, the acetone is a good solvent for the latter, and thus the collodion is exceedingly active, much more so than is the present officinal collodion of cantharides. The finished product is quite clear, and of a green colour.

ANGINA pectoris is so often associated with aortic disease, particularly aortic regurgitation, and the latter is so often due to syphilis that has been ineffectually treated in the first instance, that one is inclined to think of former syphilis almost directly one hears of a patient having attacks of angina pectoris. As a matter of fact, however, although syphilis is most assuredly one of the causes of angina pectoris, only quite a small proportion of syphilitics who die of chest complaints have had angina pectoris, and of all the patients who die after suffering from angina pectoris only a small proportion have had syphilis. The only way in which it holds good that syphilis is a cause of angina pectoris is this: that if a patient has had syphilis, treated for a short time only, the chances of his developing angina pectoris in later years are more than twice as great as they would be if he had not had syphilis at all. These statements are borne out by the statistics adduced by Professor C. J. Bouchard, of Paris, before the medical section of the British Medical Association annual meeting in the discussion upon the ætiology of degenerative changes in the aorta. Amongst 4,000 cases of chest disease in 83 anginal attacks were noted. He had expected to find that nearly all these 83 were old syphilitics, but as a matter of fact only 12 of them were. That is to say, in this series of angina pectoris cases there was a history of syphilis in only one patient out of every seven. On the other hand, the total 4,000 chest cases included 261 syphilitics, of whom only 12 had angina pectoris; in other words, although only one case of angina pectoris in seven is due to syphilis, between four and five out of every 100 syphilitics develop angina pectoris, whilst only two of every 100 non-syphilitic persons do so.

THE drug most generally employed in the treatment of the night sweats of phthisis is probably belladonna, given internally; and this same drug made up into a powder is one of the best-known external applications for that distressing normal abnormality, perspiring feet. Not only are there objections, however, to the repeated use of belladonna, but also it is not always successful in curing the complaint. It is a welcome addition to knowledge, therefore, to learn that the external application of lysol is very efficacious in the treatment both of night sweats and of perspiring feet. If the odour of the lysol is objected to, odourless nizo-phenol may be used instead. The strength employed is about a 3 per cent. solution either in plain water or else in a mixture of equal parts of water and alcohol. It may be applied either cold or warm. In the relief of night sweats the patient's body and limbs are washed with soap and water, and then sponged for four or five minutes with the lysol solution. The latter is allowed to dry spontaneously as the patient is lying between dry blankets. The application is often followed by several weeks' relief, when it has been repeated on five or six successive nights. The procedure in the case of perspiring feet is very similar, except that a footbath is employed and the patient is able to do everything for himself.

KATZENSTEIN reports in the *Berliner Klinische Wochenschrift* some interesting research work on the defence of the stomach against auto-digestion. He found that the stomach is able to digest parts or the whole of the intestine implanted within the stomach even when the mesenteries are left attached. On the other hand portions of the duodenum and stomach wall upon implantation were found entirely intact after the animals were killed. He found further that if a slip of gastric mucous membrane is added to a test tube containing normal gastric juice and a small piece of albumen, the action of the gastric juice upon the albumen is abolished by the presence of the stomach mucosa. The author concludes from these experiments that the gastric mucous membrane contains a substance which prevents its own digestion both in the living and dead state. He thinks this substance is of the nature of an antipepsin, and he attributes the causation of gastric ulcer to a deficiency or diminution in the normal amount of antipepsin in patients affected with the disease. Acting upon these suggestions the author has devised a method of treatment by means of which this deficiency can be made good, and he claims to have obtained already very successful results, but he reserves the details of the treatment and a full report of the cases so treated for a further communication.

Dr. Granger has recently published in *Merck's Archives* an interesting report on cases of malignant disease of the tongue which have been treated by him by means of mercuric cataphoresis or ionisation. The treatment was first used by Massey in 1893. It consists in the destruction of the malignant growth with sterilisation of the surrounding healthy tissue by the oxychlorides of mercury and zinc. The patient is anæsthetised, and one or more zinc electrodes, which have been previously amalgamated, are inserted into the growth to the desired depth. These are connected to the positive pole of a source of continuous current. The negative pole is connected with a large dispersive electrode placed under the patient's back. The electrolytic action of the current causes the sodium of the sodium chloride and the hydrogen of the water present in the tissues to go to the negative pole and the chlorine and oxygen pass to the negative pole, that is to the zinc electrodes, where the oxychlorides of zinc and mercury are formed. These salts penetrate the growth and destroy it by forming dead albuminates. It is, of course, necessary that the treatment be employed early in the disease, before there is infiltration of the lymphatic channels. Dr. Granger now has nine cases on record, with no recurrence in from eighteen months to three years. The value of the treatment turns entirely on the question whether it is possible in this way to eradicate the disease in the early stages with less destruction of tissue than if the growth be removed by ordinary surgical means.

---

In a communication to the Académie de Médecine M. Paul Reynier, of the Lariboisière Hospital, gives the results of his methods of treatment in cases of puerperal fever. He emphasises the fact, which is now generally recognised, that puerperal fever is not one disease but includes many types of infection with multiple lesions of varying nature and degree, for each of which there should be a special recognised form of treatment. Gonococci, streptococci, saprophytes anaerobic and aerobic, give rise to infections which differ in their local lesions, in their general symptoms, in prognosis and in treatment. An accurate diagnosis of the nature of the infection in a particular case is therefore of the first importance. The sapræmic or saprophytic type is most clearly distinguished from the streptococcal by its tendency to remain local, and M. Reynier insists that in these cases all surgical intervention should be avoided for fear of spreading infection. He advocates therefore recourse to antiseptic injections. He finds, however, that these are often inefficacious, and he has instituted a method of treatment by means of injections of oxygen gas. The uterus is first washed out with hydrogen peroxide or iodine solution, and then the two-way sound is left in the uterus and is attached to an oxygen cylinder, and a continuous current of oxygen is passed through it. The current of gas is regulated so as to avoid all pressure. According to M. Reynier the results have been most gratifying. The temperature falls, and the local diphtheroid patches clear up rapidly. In cases of extreme gravity M. Reynier has also used venous injections of elect-argol, which have already been commented upon in these columns.

---

The recently published Report of the Fifth Meeting of Practitioners Interested in Tuberculosis, Munich, contains one or two papers of import for the general practitioner. Kuhn read a paper on the application of the familiar hyperæmia method to the treatment of consumption by means of his suction mask, when wearing which the patient inspires against resistance. He recommends it especially for young subjects. The discussion, however, was not on the whole favourable, and there was not much for him to take hold of in his reply, except that no one had reported any harm following the treatment. One or two agreed that use of the mask increases the red blood-cells; but Ritter denied that this is permanent, and doubted whether in any case it is safe to attribute therapeutic effect in tuberculosis to this condition. It may be recalled that erythrocythæmia, whether physiological at high altitudes, or from raw meat feeding, is the main point upon which advocates of these special forms of treatment of phthisis base their claims. Earlier verdicts on Kuhn's suction mask have been more favourable. An important thing is to exclude or remedy any pathological condition of the upper air passages above the larynx before employing it.

---

Some interesting subjects were discussed at the recent annual meeting of the American Orthopædic Association. Among these was the question of bony union in cases of fractures of the neck of the femur treated by what in America is known as the Whitman method. In this method the injured leg is put up in plaster in a position of abduction. Morisiani strongly advocated this method some years ago, and it is a recognised method of treatment at Bardenheuer's Clinic in Cologne; but in England it is scarcely so well known as it deserves to be, though Professor Ralph Thomson, in his recent Hunterian lectures, drew attention to the merits of the abducted position in the treatment of paracephalic and paratrochanteric fractures. Dr. Forbes laid stress on the fact that under good treatment osseous union is more common than is generally supposed—a point upon which Sir Jonathan Hutchinson insisted long ago. Other speakers drew attention to the different technique employed by different surgeons, and were inclined to think that faulty union is due mainly to faults in applying the plaster. It was pointed out that, unless carefully applied, the plaster bandages are most irksome, especially to old patients. That, however, holds only in cases where they are badly applied; with proper care the patient treated by the Morisiani-Whitman method is as comfortable as in a Hodgen splint and far more so than when fixed to a Desault. Special attention was drawn to comparative success of the method in cases of fracture occurring in old people. The method is still on its trial, but the results already achieved justify its employment in suitable cases.

---

Another interesting point was raised in the discussion on certain cases of myositis ossificans. This is the pathology of the interesting bone condition which is usually known as "late rickets." There is a tendency to deny this condition—to which attention was first drawn in England by Mr. Clement Lucas—the honour of being a distinct clinical entity. It is said to be a condition of juvenile osteomalacia or osteosathyrosis, and the question has been lately reopened by Madelung, who has described cases of bending of the long bones associated with multiple subluxations occurring in adolescent patients. There is at present no possibility of deciding between the different theories. One section holds that late rickets is recrudescent rickets, while another definitely classes it as a separate disease. To the latter belongs Dr. Blanchard, who opened the discussion, and who believes that late rickets is quite different in its nature and pathology from infantile rickets and osteomalacia. The changes in the diaphysis of the long bones are much more marked in late rickets than in the infantile form; the bone itself is friable instead of being pliant as in osteomalacia, and there is a marked tendency to eburnation where it is subjected to pressure or attrition. The question of treatment was briefly discussed, members agreeing that an osteotomy is usually unsuccessful in cases of late rickets.

Yet a third interesting point cropped up in the discussion following the reading of Dr. Lovett's paper on the early orthopædic treatment of poliomyelitis—namely, how far are the deformities of infantile paralysis preventable? Cases of complete recovery are almost unknown, and the paralysis following the acute attack is very apt to be mistakenly treated. Several of the speakers emphasised the fact, frequently overlooked, that the designation "infantile" paralysis is a misnomer; the disease may, and frequently does, occur in adults, and is then often overlooked and a wrong diagnosis is made. An interesting bit of information was supplied by Dr. Arnold, who related particulars of severe epidemics of the disease among dogs, cats, and chickens. These epidemics occurred during the wet seasons, and were very similar to those noticed among infants. Dr. Sherman deprecated the irrational use of faradism in cases of marked paralysis. The treatment, in his opinion, only irritates the child. With this expression of opinion several speakers agreed, suggesting early orthopædic treatment. Dr. Lovett, in his reply, stated that the general practitioner can do much in most cases by early recognition of the disease. The most sensible line of treatment is to empty the bowels, and not to blister the spine or irritate the patient by applying electricity. In his opinion anterior poliomyelitis is primarily due to an intestinal infection by an anærobic bacillus, probably derived from milk. After the acute stage a great deal may be done by judicious educational treatment. The child should be encouraged to attempt voluntary movements and massage should be sparingly used.

In a pamphlet of some 200 pages, Dr. Massini of Buenos Ayres, has published the results of his researches into the pathogenesis of eclampsia. After discussing the various explanations which have from time to time passed into currency, such as the nervous, renal, and uræmic theories and that of placental insufficiency, etc., the author deals at length with the question of thyroid inadequacy. The following is a summary of the general conclusions to which his researches have led him:—Absence, degeneration, and extirpation of half the thyroid in pregnant bitches, produce no pathological effect under otherwise normal conditions. Extirpation of three quarters of the thyroid, which is well tolerated in the non-gravid bitch, produces marked functional disturbance in the gravid bitch. Total extirpation of the thyroid in the gravid bitch causes violent convulsive attacks, followed by death in 24 hours after the operation. These convulsive attacks bear a marked resemblance to the spontaneous eclampsia noticed in bitches. The lesions noticed after thyroidectomy are similar to those found after death from eclampsia. Without pretending to identify the symptomatology of canine eclampsia with that of the gravid auto-intoxication of women, the similarity of the one to the other is sufficiently great to deserve attention.

Another striking fact is that, after delivery, phenomena disappear alike in the gravid woman suffering from auto-intoxication and in the bitch rendered eclamptic by thyroidectomy. It is clear that the thyroid gland is indispensable during gestation, and its absence or insufficiency causes eclampsia in the gravid bitch. The author suggests that there is a poison circulating in the blood of gravid women, the result of the penetration of the chorionic villi into the maternal blood stream. The antitoxic action of the thyroid is usually sufficient to neutralise the poison, but when the gland is deficient either as the result of pathological change or of operative interference, the poison circulates without restraint, and leads to eclampsia.

A paper by Drs. Quenu and Kuss, read before the Société de Chirurgie, discusses the nature of the jaundice sometimes seen after operations under chloroform. The authors attribute this jaundice to alterations in the liver-cell produced by the chloroform circulating in the blood. Certain clinical peculiarities permit this form of jaundice to be differentiated from others. It is usually the result of a long narcosis during which the patient has received a large dose of chloroform. It frequently commences with symptoms of renal and hepatic insufficiency, such as somnolence, cerebral phenomena, and later coma, oliguria, and sometimes albuminuria; and it generally ends in death. This form of jaundice occurs for the most part in patients who are already suffering from some form of hepatic disease, recent and old lesions of the liver being found side by side at the post-mortem examination. Jaundice due to chloroform poisoning is thus comparable to that due to poisons which act mainly on the liver—*e.g.* phosphorus. But chloroform has also a distinct hæmolytic action, so that jaundice is sometimes seen in subjects who have no appreciable liver disease. This form of jaundice, however, is slight and unaccompanied by hepatic or renal symptoms, and it passes off in two or three days.

